# Medical News and Miscellany

**Published:** 1896-10

**Authors:** 


### Medical News and Miscellany.

**Dr. A. O. Trenckmann,** has removed from New Ulm to Bellville, Texas.

**Dr. E. M. Albers** has removed from William Penn to Victoria, Texas, his former home.

**Wanted.**—Gynecological chair. Must be in good condition. Cheap for cash. Write full particulars to P. O. Box 78, Victoria, Texas.

**Prof. J. H. Callender,** the noted alienist and neurologist, of Nashville, died recently in that city.

---

**The** JOURNAL learns that Dr. J. C. B. Renfro has removed from Houston to La Grange and formed a copartnership with Dr. F. A. Schmitt, of that place. Dr. Renfro formerly resided at La Grange, we believe.

---

**Prof. Hodge's** paper on the effects of the bromides, published in this issue should be read by all. It will create surprise in the minds of some who have for years thought the bromide of potassium a perfectly harmless remedy—except as that it produces in some persons a rash on the skin. It is a timely note of warning which it would be well to heed.

---

**Many** of our subscribers have *requested* us to draw on them for subscription. On the other hand some subscribers have taken deadly offense at being drawn on. We want to say that we will send out every bill on the books with this issue; and subscribers in arrears may expect a draft unless we hear from them in due time. It is *business*: we need our money: we have given long indulgence, and there is no offense meant. Pay the draft if convenient, if not, do not get offended.

---

**Dr· Bibb.**—By reference to the advertisement of the M. C. R. R. in this issue, it will be seen that special arrangements have been made for the delegates to the Pan American Medical Congress to be held in the City of Mexico, November 16–18. Dr. R. H. L. Bibb, the chief surgeon of the M. C. R. R.,—whom everybody knows,—an old Texas practitioner, and one of the most popular men in the profession, will meet the excursion at Laredo and take personal charge of the delegates and their friends. Dr. Bibb is excellent company, and will make an admirable host. If all the delegates do not have a good time it will not be the doctor's fault.

---

**A Request.**—To the profession of Texas: The success attending the efforts of the profession of Fort Worth—unaided—to establish a high grade medical school has been as remarkable as it has been gratifying. The school is a credit to the State, as well as to those connected with it. They have never asked aid of any one. Now that it is able to take care of itself the management feel that they can call on professional friends throughout the State to lend or donate to the museum specimens of interest, curios, relics, etc. All such contributions will be gratefully acknowledged. They will be properly labeled indicating owner or donor. Short history should accompany each specimen, and they should be sent to Curator of Museum, Medical Department, Fort Worth University.

---

**Nothing New.**—Brown-Sequard made much notoriety and got great credit for suggesting the testicular juice cure for certain ailments. An esteemed correspondent calls our attention to the fact that the treatment is as old as the hills, and cites us to the oldest editions of Dunglisson's dictionary for an account of it. It is also in the newer editions. Under the head of *Phasianus Gallus* is the following: "The parts of generation of the cock, dried and pulverized, were formerly regarded as proper for increasing the quantity of semen. The fat was used as emolient and resolvent; the brain in diarrhœa, the gall in freckles and diseases of the eye. These fantasies are now abandoned." (The word is derivd from the Greek "*phasialos*," the river Colchis, near the Black Sea, and means "cock;" French, coq.

---

**The Charlotte, N. C., Medical Journal,** now in its fifth year, is a model medical journal and a marvel of mechanical execution. Its success, too, has been commensurate with its merit, a rare exception,—and more than seven thousand physicians have testified their appreciation by subscribing for it. Drs. Register and Montgomery, the owners and editors, are to be congratulated both upon the excellence of their work and upon the pecuniary success attending it. They have recently purchased and put in operation a special plant for the publication of the *Journal* alone, at a cost of $10,000. Those of our readers who have not seen the *Jouanal* would do well to write for a specimen copy. The subscription price is $2.50 a year.

---

**The Doctor Remembered.**—The life of the doctor is not one of ease, and his pathway is not strewn with flowers. Too often it is a thankless mission. But, fortunately, there are rare instances of real appreciation of the devoted doctor's services that shine out with great lustre on account of the rarity; and it is always a satisfaction to record them. But when the good luck falls to a personal friend, and a well-known doctor, to be made the recipient of handsome evidences of appreciation at the hands of the well-to-do whom he has served, it affords us more than ordinary gratification to record the fact. We learn that Ex-State Health Officer R. Rutherford, of Houston, the long-time physician to the decedent, was handsomely remembered in the will of old lady Rice lately; she bequeathing the doctor twenty-five thousand dollars out of her large estate, in testimony of appreciation of his services. The fortunate doctor has the JOURNAL's sincere congratulations.

---

**Convicted of Abortion.**—An associated press dispatch to the Austin *Statesman*, September 10, ult., states that "Dr. L. B. Miller, charged with abortion on Miss Allie Turnidge, whom he afterwards married under pressure, but refused to live with, was convicted in the criminal district court, and given seven years in the penitentiary."

Here is a case for Dr. Daniel's remedy: Castration. Abortion alone, under ordinary circumstances, is bad enough; but seduction *and* abortion constitute a crime that deserves something better than penitentiary; castration only will meet the requirements in such a case; and a "doctor" too! Alas, our "noble profession."

---

**Wait Till the Clouds Roll By.**—Never before, perhaps, has there been such *unrest* in the ranks of the Texas profession; never so much moving about, and desire to change location. We have numerous requests to find purchasers for practices,—(or for real estate, practice thrown in,) and also numerous inquiries for locations. In almost every instance where one has not done well recently, there is a disposition to hunt a better place. The JOURNAL's advice to all is, to let well enough alone; if one has made expenses the last three years, he had better stay where he is, than to flee to other fields he knows not of; he might jump out of the frying pan into the fire. It is expensive to move; and when a doctor leaves a place, he may as well burn his books and accounts, for few will pay for past services when no future service is in sight. Thus is added a heavy loss to the expense of moving. A doctor is the last person in the world who ought to move. When a doctor locates, he should select his location with great discretion, and stay there. If he remains, say five years, and moves away, the fives years are practically lost; he begins over,—and has to go through the same process; in the meantime, he is growing older. Times are hard everywhere, and one can hardly better himself by a change of location. Stay where you are and wait for the reaction, which is sure to come.

---

**College Changes.** — Dr. H. F. Harris, long time professor of chemistry and clinical medicine in the Southern Medical College at Atlanta, has accepted the position of associate professor of bacteriology, in the Jefferson at Philadelphia, and Dr. A. M. Purse has been appointed to succeed Dr. Harris; and Dr. G. G. Roy, professor of materia medica in the Southern, has been succeeded by Dr. C. D. Hurt—Dr. Roy remaining as emeritus professor. On the death of Dr. Powell, the founder of the school, Dr. W. Perrin Nicolson, the dean—was made president of the college by the unanimous vote of the trustees. Dr. J. G. Bourns, late of Ann Arbor, has been elected professor of bacteriology and pathology, and Dr. Lucien Lofton, of Atlanta, has been appointed assistant to the chair of anatomy and assistant demonstrator of anatomy.

The Atlanta Medical College has made the following changes: Dr. H. P. Cooper will succeed Dr. W. S. Armstrong, lately deceased, as professor of anatomy. Dr. Hubbard, of Atlanta, appointed assistant to the chair of materia medica.

---

**Another Doctor Killed.**—On the 13th of September, ult., Dr. W. M. Drake, a prominent physician of Hillsboro, Texas, was shot and killed by Mrs. Roberts, wife of a Doctor Roberts, a drug clerk in Hooper's drug store. Dr. R., who also took a hand, was wounded. The facts are hard to get at, but from the best information to be had it seems that Mrs. Roberts informed Dr. Roberts, the husband, that Drake had insulted her. Dr. and Mrs. R. drove up to the drug-store in a buggy, and the doctor getting out, soon engaged in a rough and tumble fight with Drake, who was standing on the sidewalk. It was a dog-fall; but Drake got on top, and was in the act of disarming his antagonist, when guns began to go loose. Drake was killed, but he got in one shot on Roberts before he yielded up the ghost. Mrs. Roberts was arrested, and is held on the charge of murder or manslaughter. Dr. Drake formerly lived at Abbott. He was a graduate of Columbus, O., Medical College of class of 1881, and was at one time a member of the Texas State Medical Association.

GALVESTON, TEXAS, August 27, 1896.

*To Members of the Texas State Medical Association:*

I have been requested by Dr. C. A. L. Reed, of Cincinnati, O., upon authority of the International Executive Committee of the United States, to give notice that I will issue delegates' certificates to such members of our State Association as may apply to me for the same to the Second Pan-American Medical Congress, to be held in the City of Mexico, November 16 to 19, inclusive, 1896. With the understanding that I am authorized to do so, it will give me pleasure to furnish such credentials to all members in good standing who may wish to attend.

Fraternally yours,

H. A. WEST, Secretary,
2020 Market Street.

---

**Alas! Poor Texas.**—The Lone Star State is made the scape goat for all kinds of falsehoods and ridicule. Here is the latest:

"Effective Health measures in Texas.—A veracious Englishman, living in Texas, wrote as follows to his mother, who lives near Gloucester, in England: 'I have been much interested in the accounts of the small-pox in your neighborhood. In this free and enlightened country, when it broke out in a town every one was ordered to be vaccinated. Those who objected were held against a wall by one policeman, and another stood opposite with a loaded revolver while the operation was being performed. I should much like to assist in the same way at the vaccination of some of the Stroud people.' The letter was printed in a local paper, and now the good people of the place are full of admiration of the way our sanitary laws are enforced."—*New York Medical Record.*

---

**The publication**, *Climate and Health*, a monthly journal issued by the Weather Bureau of the Agricultural Department, has been discontinued. The last number, No. 3, Vol. II, for the four weeks ending March 28, 1896, was issued June 22d. Much good was expected to come of this publication. It was an effort to record statistics and data pertaining to climate and its relation to hygiene—from which might be learned the influence of climate upon health, and consequently furnish indications for the proper treatment of certain diseases, and it is to be regretted that the enterprise had not the necessary backing of government to carry it through. The undertaking met with earnest co-operation of medical men interested in that branch of study, but red tape, it seems, terminated its brief career. "A doubt," says the *Journal of the American Medical Association*, "as to whether the publication was authorized by the act making appropriation for the Department of Agriculture for the fiscal year ending June 30, 1897, was the cause of its discontinuance." Professors Hamilton and Moore, and Dr. Philips, who edited the journal, deserve credit for their enterprise and the excellent work put upon the few numbers that saw daylight.

---

**The Pasteur Institute** at Chicago. Dr. A. Lagorio, director of the Chicago Pasteur Institute, has issued his annual report. He says:

"A total of 532 patients have been treated to date at the Chicago Pasteur Institute since its inauguration, July 2, 1890.

"The patients treated have been divided into three classes: 1. Those bitten by animals recognized and ascertained to be rabid by the control experiment made in the laboratory, or by the deaths of other persons or animals bitten by the same animal. Of these 183 were treated. 2. Those bitten by animals recognized to be rabid by the symptoms of rabies shown during life. Of these 237 were treated. 3. Those bitten by animals strongly suspected to be rabid. Of these 112 were treated. Grand total, 532.

"When we consider the high percentage of deaths occurring previously to the discovery of the Pasteur treatment, which was 88 per cent. for the bites of the head and face, 67 per cent. for the bites of the hands, and 20 to 30 per cent. for the bites of limbs and trunk, we have reason to congratulate for the excellent results attained at this institute, as only two deaths have been reported, giving a mortality of 0.37 per cent., thus proving, without question, the efficacy, as well as the harmlessness, of the Pasteur anti-hydrophobic treatment.

"483 persons were bitten by dogs, 24 by cats, 13 by horses, 5 by skunks, 2 by wolves, 1 by a mule, 1 by a pig, and 2 by hydrophobic human beings.

"254 persons received severe and multiple bites on the hands and wrists, 62 on the head and face, 70 on the arms, 119 on the legs and thighs, and 27 on the trunk.

"The patients came from the following States: 294 from Illinois, 41 from Iowa, 41 from Ohio, 36 from Indiana, 38 from Kansas, 11 fiom Arizona, 12 from Minnesota, 8 from Missouri, 6 from Kentucky, 5 from Tennessee, 5 from Louisiana, 4 from Michigan, 6 from Nebraska, 5 from Texas, 3 from Wisconsin, 3 from Indian Territory, 2 from South Dakota, 1 from North Dakota, 1 from New Mexico, 1 from Arkansas, 1 from Pennsylvania, and 1 from Oklahoma Territory.

"410 other persons applied for the Pasteur treatment, but were sent back, as it was recognized that the animals which inflicted the bites were not rabid.

